# Beyond acute infection: molecular mechanisms underpinning cardiovascular complications in long COVID

**DOI:** 10.3389/fcvm.2024.1268571

**Published:** 2024-02-14

**Authors:** Roba Hamed Mostafa, Ahmed Moustafa

**Affiliations:** ^1^ Systems Genomics Laboratory, American University in Cairo, New Cairo, Egypt; ^2^ Biotechnology Graduate Program, American University in Cairo, New Cairo, Egypt; ^3^ Department of Biology, American University in Cairo, New Cairo, Egypt

**Keywords:** SARS-CoV-2, cardiovascular, long COVID, post-acute COVID-19 syndrome (PACS), transgenerational inheritance

## Abstract

SARS-CoV-2, responsible for the global COVID-19 pandemic, has manifested significant cardiovascular implications for the infected population. These cardiovascular repercussions not only linger beyond the initial phase of illness but have also been observed in individuals who remain asymptomatic. This extended and pervasive impact is often called the post-acute COVID-19 syndrome (PACS) or “Long COVID”. With the number of confirmed global cases approaching an alarming 756 million, the multifaceted challenges of Long COVID are undeniable. These challenges span from individual health complications to considerable burdens on worldwide healthcare systems. Our review comprehensively examines the complications of the persistent cardiovascular complications associated with COVID-19. Furthermore, we shed light on emerging therapeutic strategies that promise to manage and possibly mitigate these complications. We also introduce and discuss the profound concerns regarding the potential transgenerational repercussions of SARS-CoV-2, emphasizing the need for a proactive and informed approach to future research and clinical practice.

## Introduction

In December 2019, Wuhan in China experienced a Coronavirus Disease 2019 (COVID-19) outbreak, leading to a global pandemic. This highly infectious virus, the severe acute respiratory syndrome coronavirus (SARS-CoV-2), primarily invades the respiratory system, causing severe pneumonia that can progress to acute respiratory distress syndrome (1). By February 2023, the World Health Organization (WHO) had reported approximately 756 million confirmed cases of COVID-19 globally, along with a staggering 6.8 million cumulative deaths attributable to the virus (2).

The COVID-19 virus contains five structural proteins: spike (S), envelope (E), membrane (M), and nucleocapsid (N). The distinctive crown-like morphology of the coronavirus results from the spike protein, which also facilitates host cell entry and consists of S1 and S2 subunits (3).

SARS-CoV-2, part of the Beta-CoV genus and the Nidovirales order, belongs to the Coronaviridae family. Notably, two previous beta-CoV virus outbreaks—SARS-CoV and Middle East Respiratory Syndrome (MERS) viruses—hit the world in 2002 and 2012, respectively (3).

SARS-CoV-2 has shown higher virulence than SARS-CoV, which is attributed to two key acquired mutations. The receptor binding domain (RBD), a highly variable segment in the SARS-CoV and SARS-CoV-2 genomes, uses six central amino acid residues for adequate recognition of the host angiotensin-converting enzyme 2 (ACE2) receptor. In SARS-CoV-2, mutations in five of the six primary amino acid residues in the RBD allow for a greater binding affinity to the ACE2 receptor. Moreover, an insertion mutation at the S1/S2 fusion site introduces a furin cleavage site, enhancing cellular entry and viral tissue tropism due to S-priming with furin and other proteases (4, 5).

SARS-CoV-2 has led to a significant cardiovascular burden in the infected population, with harmful cardiovascular sequelae persisting even after the acute phase and in asymptomatic patients. This review focuses on the pathophysiology underlying the long-term cardiovascular burden associated with COVID-19 and explores potential new therapeutic targets.

## COVID-19 pathogenesis and pathophysiology

Host neuropilin-1 (NRP-1) binding to the NRP-1 binding domain in RBD precedes ACE2 recognition. This action facilitates host ACE2 recognition by the RBD of COVID-19, triggering a conformational change that allows the virus to become closer to the host cells (6).

SARS-CoV-2 can enter cells through one of two pathways: membrane fusion or endocytosis. In membrane fusion, a host protease known as transmembrane protease serine 2 (TMPRSS2) cleaves and primes the spike protein. Subsequently, the fusion peptide within the S2 subunit is inserted into the host cell membrane. The two heptad repeat domains in the S2 subunit, HR1, and HR2, interact, drawing the viral and host membranes together and enabling membrane fusion. This process allows the injection of the viral genome into the host cell. In the endocytosis pathway, the virus-receptor complex is endocytosed into the endosome, where the S protein is cleaved by cathepsin enzymes (7–9).

SARS-CoV-2 exhibits a broad tissue tropism, as its receptor is expressed in various tissues, including the lung, heart, small intestine, oral mucosa, and testis. These tissues have been found to harbor SARS-CoV-2 RNA (10–13). Viral entry triggers multiple immune responses. Toll-like receptor 3 (TLR-3) recognizes the cytosolic viral genome and activates nuclear factor kappa B (NF-κB), which translocates into the nucleus and stimulates the transcription of interferon-gamma and other inflammatory cytokines. Additionally, the NOD-like receptor (NLRP) gene is activated, leading to the production of NLRP3 proteins. NLRP3 initiates the formation of inflammasomes, which induce cell death through pyroptosis and promote inflammation by stimulating the secretion of inflammatory cytokines (14, 15). This immune response activates a wide range of immune cells, including dendritic cells (DCs), *T*-cells, and B-cells. DCs carry the viral antigen with major histocompatibility complex class II (MHC-II) receptors and migrate to lymph nodes to stimulate further *T* and B cell responses. *T*-cells and B-cells are then activated to eliminate the virus through direct cell killing and antibody secretion, respectively (14, 16).

The excessive release of inflammatory cytokines often leads to a cytokine storm, a lethal state of hyperactive immune response associated with systemic inflammation. Persistent systemic inflammation can cause vasculitis, increasing the risk of blood clot formation, hypercoagulability, ischemia, and cell death. Additionally, acute respiratory distress syndrome results in poor perfusion, hypoxia, multiorgan failure, and death (14, 16).

Although COVID-19 was initially considered primarily a respiratory illness, it is now evident that it causes a systemic infection, damaging multiple organs during and after the infection. This damage may arise from a direct viral infection or the detrimental effects of systemic inflammation (17).

## COVID-19 and the cardiovascular system

Cardiovascular injury is common among SARS-CoV-2-infected patients, with damage to the myocytes varying from initial injury with elevated troponins to eventual heart failure, indicated by increased levels of the N-terminal-prohormone brain natriuretic peptide BNP (NT-proBNP) (18–20). Among the cardiovascular complications linked to COVID-19 are arrhythmia, myocarditis, acute coronary syndrome, myocardial infarction, and venous thrombosis embolisms, which are detectable through methods such as echocardiography, MRI, electrocardiogram (ECG), coronary angiography, and cardiac autopsies in COVID-19 patients (20–22).

Cardiac involvement in COVID-19 patients predisposes to a higher mortality rate compared to COVID-19 patients without such complications. Higher troponin levels in COVID-19 patients with pre-existing cardiovascular diseases have been associated with doubling the mortality rate (18, 23). Children may also suffer from cardiac dysfunction and coronary abnormalities, such as Kawasaki disease (24).

## Mechanisms of COVID-19-associated cardiovascular injury

Although several mechanisms have been proposed, the precise pathophysiological pathway by which SARS-CoV-2 causes cardiac damage has not been entirely established. One such pathway involves SARS-CoV-2-induced hypoxemia secondary to pulmonary dysfunction and respiratory distress syndrome. This results in poor organ perfusion, leading to hypoxic injury in cardiac cells and contributing to myocardial infarction. Another related mechanism involves microvascular injury and thrombosis in the lung microvasculature, which leads to right ventricle heart failure (7, 25).

Systemic inflammation and cytokine storms play a vital role in microvascular injury, endothelial cell activation, venous thrombosis, and the hypercoagulative state associated with COVID-19 (26). This concurs with findings from heart autopsies of COVID-19 patients, where venous thrombosis and immune cell infiltration by CD3+ and CD8+ cytotoxic lymphocytes, CD68+ macrophages, and CD45RO memory cells were identified (27–29). Overexpression of cytokines can cause myocarditis and myocardial injury (27, 30), and cardiac biomarkers have been found to correlate with the levels of inflammatory cytokines (26, 31). Moreover, patients with predisposing cardiovascular risk factors are more susceptible to microvascular injury and endothelial dysfunction since SARS-CoV2 associated inflammation exacerbates the pre-existing endothelial damage aggravating thrombosis which leads to an increased risk of acute coronary syndrome and myocardial injury (32).

Direct virus entry is another proposed mechanism of COVID-19-associated cardiac damage. Inflammation and oxidative stress have also been shown to increase the entry of the SARS-CoV-2 virus into cardiac cells and promote cellular apoptosis (33). Linder et al. documented the presence of SARS-CoV-2 RNA in 61.5% of myocardial autopsies of patients who died of COVID-19, with the viral load exceeding 1,000 copies per μg RNA in 41% of the tissues (27). On the other hand, another study denied the existence of viral RNA and recorded very few RNA copies (34, 35). Similarly, Moustafa et al. found that traces of SARS-CoV-2 RNA are present in peripheral blood mononuclear cells of COVID-19 patients, although in significantly lower quantities compared to bronchoalveolar lavage specimens, suggesting limited viral RNA in the blood (36). Han et al. suggested that COVID-19 could directly infect the heart's pacemaker, causing arrhythmia in hamster models and human embryonic stem cell (hESC)-derived SAN-like pacemaker cells, where the viral RNA was detected inside the pacemaker cells and was capable of inducing ferroptosis (37). In Drosophila and mouse models, non-structural protein 6 (NSP6) of SARS-CoV-2 interacted with host proteins in the heart, potentiating glycolysis, disrupting mitochondrial function, and increasing ROS formation (38).

After viral entry, COVID-19 patients are susceptible to hypertension due to the downregulation of ACE2 receptors in host cells. The ACE2 receptor plays a critical role in the renin-angiotensin-aldosterone (RAAS) system, with its downregulation leading to the upregulation of angiotensin 2, a potent vasoconstrictor (10, 14, 16).

Recent studies using omics-based analyses have provided a more comprehensive understanding of the pathophysiology underlying COVID-19-associated cardiovascular diseases. In the heart tissues of COVID-19 patients, there was an observed upregulation in the transcriptional level of phospholipase C γ2 (PLCG2) in pericytes, fibroblasts, and cardiomyocytes. In contrast, the Afadin level, encoded by the Adherens Junction Formation Factor (AFDN) gene, was predominantly high in endothelial cells (34). Although the mechanism of PLCG2 in the heart is not thoroughly investigated, PLC-γ is known as a regulator of calcium homeostasis within heart tissues and plays a crucial role in cardiac pathogenesis. Its upregulation has been associated with cardiomyocyte apoptosis and mitochondrial dysfunction in patients with myocardial infarction, while its dysregulation can cause cardiac hypertrophy (39, 40). Furthermore, PLC is proposed as an arrhythmogenic factor, exacerbating cardiac fibrosis (41, 42). Whereas Afadin is essential for maintaining endothelial barrier function, the mechanism by which it is linked to SAS-CoV2-associated CVD is not known. Enrichment analysis revealed upregulation in cell differentiation, cell adhesion with elevated immune response, and apoptosis due to oxidative stress in fibroblasts, cardiac cells, and pericytes, respectively (34).

Meanwhile, endocrine senescence pathways in plasma were significantly upregulated in COVID-19 patients with cardiac complications. High FSTL3 and low ADAMTS13 levels were associated with heart failure in COVID-19 patients. The former is an indirect marker of biological aging induced by the TGF-B pathway, and the latter is an antithrombotic agent (19, 43). Another study by Garg et al. found elevated levels of miR-155, miR-499, miR-208a, miR-21, and miR-126 in the blood of critically ill COVID-19 patients. These miRNAs are associated with inflammation, myocardial function, fibroblasts, and endothelial cells (44).

Lipopolysaccharide (LPS)-binding protein (LBP) was upregulated and strongly associated with NT-pro-BNP in critically ill patients with cardiac involvement. LBP levels increase in response to circulating LPS, a major component of the outer membrane of gram-negative bacteria, suggesting gut leakage as an additional mechanism that could exacerbate heart failure in SAR-CoV-2-infected patients (45–48).

## Cardiovascular manifestations of long COVID

Recently, it became evident that the adverse effects of COVID-19 are not limited to the acute phase but also extend to a condition known as long COVID. According to the National Institute for Health and Care Excellence (NICE), patients with ongoing symptoms of COVID-19 or newly developed ones that last beyond three months are diagnosed with long-term COVID (49).

In the Netherlands, patients were followed for three months post-infection and showed evidence of elevated inflammatory cytokines and endothelial dysfunction. This is in line with the elevated plasma levels of endothelin-1 (ET-1) that correlate with contact activation factors, hence suggesting endothelial activation and the coagulation process (50). In England, hospitalized COVID-19 patients suffered from an increased rate of major adverse cardiovascular events after discharge. People without any prior cardiovascular events developed cardiovascular diseases at a rate three times higher compared to healthy individuals (51). Another study followed patients for four months post-infection, and the rate of various cardiovascular diseases increased with new onsets of hypercoagulability and cardiomyopathy sequelae post-infection (52). Similarly, in the USA, a substantial excess burden of cardiovascular diseases occurred in a graded fashion according to the severity of the acute phase when patients were followed in two studies for six months and a year (17, 53). The commonly detected long-term cardiovascular sequelae were cerebrovascular disorders, dysrhythmia, pericarditis, myocarditis, ischemic heart diseases, hypertension, heart failure, and thrombotic disorders (17, 52, 53).

## Pathophysiology of long COVID cardiovascular complications

**Figure 1:**
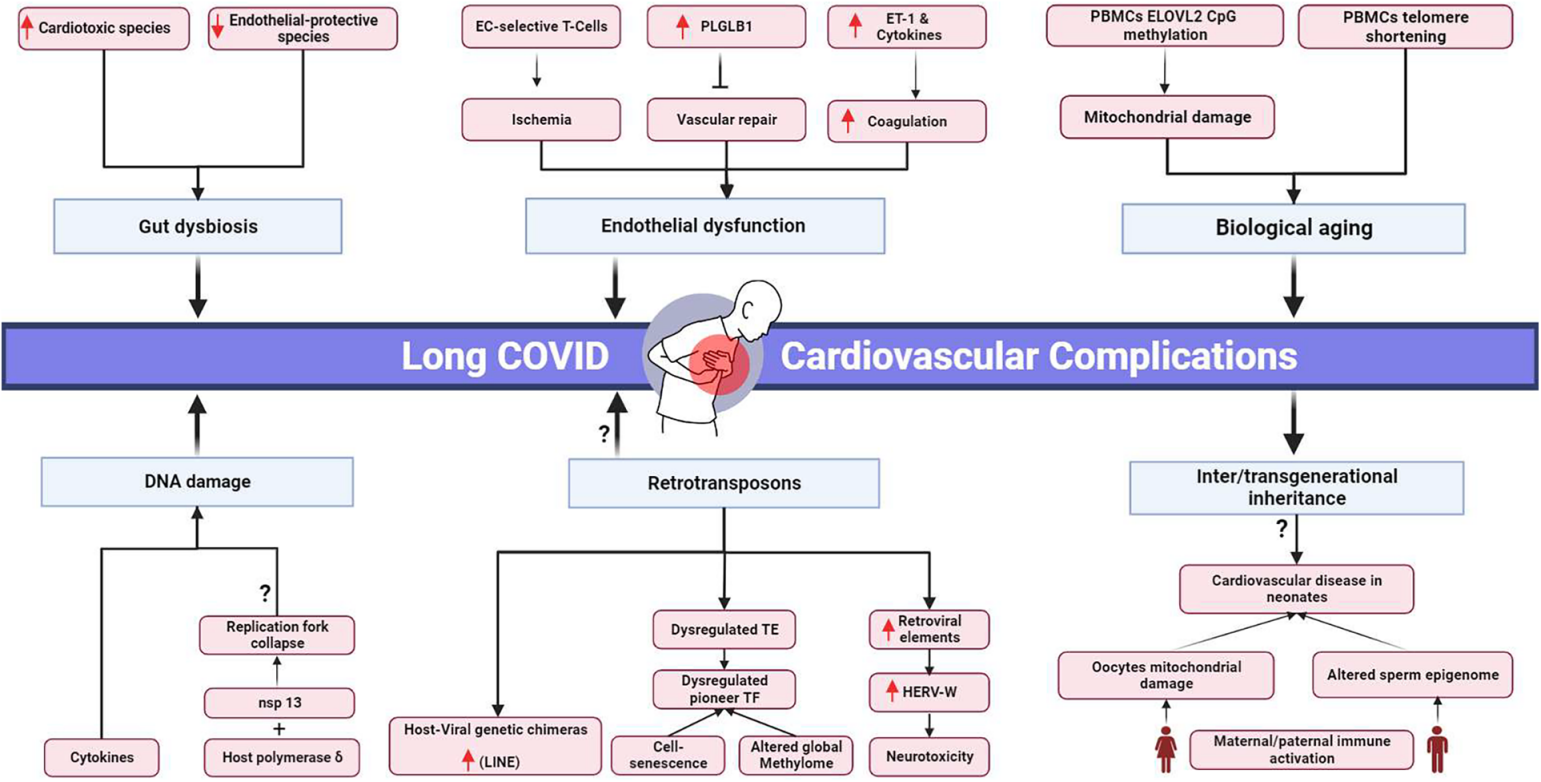
Proposed pathophysiological mechanisms and potential transgenerational cardiovascular impact of long COVID. EC, endothelial cells; PLGLB1, plasminogen-like protein B1; ET-1, Endothelin-1; PBMCs, peripheral blood mononuclear cells; *ELOVL2*, elongation of very long chain fatty acids-like 2; nsp 13, non-structural protein; HERV-W, human endogenous retrovirus-w. Black arrows represent downstream sequel, bars represent inhibitory downstream sequel, red arrows represent upregulation, question marks represent hypothesized mechanisms requiring further research. Created in BioRender.com.

Long COVID's clinical burden stresses healthcare systems and demands resources (54), emphasizing the urgency to understand the clinical pathology behind the disease and identify novel clinical targets (Figure 1). However, the absolute mechanism underlying long COVID sequelae has yet to be extensively deciphered.

### Cell senescence and mitochondrial dysfunction

The first proposed mechanism is accelerated biological aging. In COVID-19 survivors’ peripheral blood mononuclear cells (PBMCs), the cells were five years above their expected biological age (55). Furthermore, accelerated aging was associated with downregulated ACE2 levels and telomere shortening, which are notable in cardiovascular diseases (56, 57). The senescence-associated secretory phenotype was reported to be correlated with cardiac involvement in COVID-19 patients (19).

Mitochondrial damage is another cornerstone of biological aging (58). In COVID-19 post-acute phase survivors, there was considerable CpG island methylation in the promotor region of elongation of very long chain fatty acids-like 2 *(ELOVL2)* in PBMCs (55). ELOVL2 is crucial for proper mitochondrial function. Mitochondrial dysfunction in COVID-19 patients’ PBMCs was reported by Ajaz et al. (59). PBMCs with damaged mitochondria are known to aggravate heart failure (60, 61). Another study found downregulated profiles of genes responsible for metabolic and mitochondrial function in SARS-CoV-2-infected cardiac tissues (62). In the iPSC-derived human cardiomyocytes (iPSC-CMs) model, SARS-CoV-2 infection impaired mitochondrial function, cell bioenergetics, and calcium cycling (63).

### DNA damage and cell cycle arrest influenced by SARS-CoV-2

DNA damage presented another pathogenic pathway inhibiting the symptoms’ resilience in COVID-19 patients. However, further studies are needed to investigate the longevity of these genetic instabilities and their correlation to long COVID. DNA damage was detected in cultured AC16 cardiomyocyte cells treated with the serum of COVID-19 patients, where markers of DNA double-strand break, γH2Ax, and H3K79me2, were elevated. Persistent inflammatory cytokines were detected in long COVID patients’ serum samples, which are inducers of chromosomal instability (64–66). In agreement, γH2Ax was upregulated in cardiac autopsy tissues from COVID-19 patients (62). SARS-CoV showed an increase in H2AX histone phosphorylation in the infected population due to the interaction between its nonstructural protein nsp13 and the host DNA polymerase *δ*, leading to replication fork collapse and DNA damage followed by cell cycle arrest. SARS-CoV-induced cycle arrest led to the consumption of host cellular metabolites to allow viral replication (67). Considering the 79% similarity between SARS-CoV and SARS-CoV-2 and the 99.8% similarity between nsp13 in both species (64, 68, 69) and that SARS-CoV-2 can directly infect myocardium and actively replicate inside (27), this could be a potential mechanism by which cardiomyocyte damage occurs during COVID-19 and extends towards long COVID manifestations.

### Prolonged immune activation and endothelial damage

Another potential molecular pathway for long COVID is endothelial dysfunction, leading to ischemic injuries and organ damage. Prolonged active CD8+ and CD4+ effector *T* cells selective for endothelial cells were detected in the blood of COVID-19 convalescent patients (70). In another study, the anti-angiogenic plasminogen-like protein B (PLGLB1) protein was more elevated in the serum of convalescent patients than in the acute phase. PLGLB1 inhibits vascular cell proliferation and prevents vascular repair in damaged vessels due to SARS-CoV-2 infection. Higher PLGLB1 levels in convalescence than in the acute phase indicate endothelial deterioration (71, 72).

Moreover, the immune system remains active in COVID-19 long-haulers, where serum samples showed dominant alterations in type II interferon and NF-κB signaling pathways (65). In PBMCs, miR-155-5p was upregulated during the post-acute phase, which is a factor known to be associated with inflammation and cardiovascular diseases (73, 74).

### Retrotransposons and genetic reprogramming in myocytes

SARS-CoV-2 has been implicated in entering heart cells and integrating its reverse-transcribed genome into the host cell genome. In a study where SARS-CoV-2 infection was introduced into human iPSC-derived cardiac cells, chimeric viral-host transcripts were detected, suggesting the possibility of integrating the viral genome into the host’s genetic material. This mechanism is hypothesized to be driven by the long interspersed nuclear elements (LINE-1) retrotransposition mechanism (75). However, more research is required to confirm this hypothesis, which is considered a rare event. Notably, an independent study suggested that these chimeric transcripts might be artifactual and created during the preparation of RNA-seq libraries via RT-switching (76, 77).

An increase in the retrotransposon element (LINE) was observed in the lungs and intestines following the SARS-CoV-2 infection. This surge was attributed to an increase in ten-eleven translocation (TET) enzymes, which alter the methylation profile and activate LINE transcription (78). Transposable elements (TE) were found to be upregulated in bronchoalveolar biopsies but downregulated in PBMCs. The downstream targets for these dysregulated TEs were enriched for pioneer transcription factors (TF) and immune responses. Pioneer TF can alter the global methylome profile of the cells. Compared to other viral infections, TE levels in COVID-19 showed the highest copy number (79). The retroelements are also known to be associated with cell senescence and aging (80, 81). Further studies are needed to analyze their levels in correlation to SARS-CoV-2 long-term cardiovascular complications.

Additionally, the Human Endogenous Retrovirus-W (HERV-W) protein, known to be stimulated in viral infection and to induce immune and neurotoxic deleterious effects, was found elevated in the endothelial cells and pericardial fatty tissues of postmortem cardiac autopsy samples of COVID-19 patients (82–84).

### Altered gut microbiome

The gut microbiota has been implicated in the pathophysiology of various diseases, including COVID-19. During the acute phase of COVID-19, the gut leakage marker Lipopolysaccharide Binding Protein (LBP) was found to be elevated in plasma samples and was associated with cardiovascular complications via the activation of inflammasomes (46, 48). Persistent gut dysbiosis was found in hospitalized COVID-19 patients, and this imbalance between opportunistic pathogens and beneficial symbionts prolonged after the acute phase (85–87).

In a 6-month period of COVID-19, a significant difference in gut microbiota composition was observed between patients with long COVID symptoms and those without symptoms or healthy individuals (88). *Ruminococcus gnavus* was found to be elevated in post-acute COVID-19 syndrome (PACS), known for its association with atherosclerosis and coronary artery disease. *Faecalibacterium prausnitzii*, a protective species against atherosclerosis, was depleted in PACS patients. *Collinsella aerofaciens* level was low in PACS, and its decreased level was associated with CAD and cardiac valve calcification (88–94). Furthermore, *Bifidobacterium pseudocatenulatum*, known to ameliorate TNF inflammatory signals and protect against endothelial damage, had an inverse relationship with PACS symptoms. On the other hand, Bacteroides vulgatus, an atherosclerosis attenuator species, was found to be abundant in PACS patients (88, 95–98).

## Intergenerational and transgenerational inheritance

Long-term cardiovascular effects of COVID-19 have been observed in the infected population. However, whether these effects extend to future generations is still unknown (17, 53). Systematic reviews suggest that 20%–30% of neonates born to SARS-CoV-2-positive mothers are infected due to vertical transmission, exhibiting clinical manifestations such as hypotension and tachycardia (99, 100).

Neonates born to positive mothers or preconception-positive parents are still at high risk of developing adverse events, even if they are not infected. Growing evidence shows that parental environmental factors, stress, and infection before or during conception can influence the offspring's phenotype by modulating their epigenetics (101–103). These epigenetic markers can be inherited by the offspring or grandoffspring, suggesting a potential role for the intergenerational and transgenerational inheritance of SARS-CoV-2 adverse events due to parental exposure to SARS-CoV-2 (102).

In the context of paternal contributions, infection and immune activation can alter the sperm epigenome and affect future generations. The sperm transfers its epigenetic information into the embryos, impacting embryonic development (104–106). Moreover, cardiovascular disease inheritance by sperm non-coding RNA was suggested by Wagner et al. (107). There is mounting evidence that SARS-CoV-2 negatively affects sperm, altering the semen proteomics of convalescent patients, which is associated with multiple pathways, including inflammatory cytokines, sperm differentiation, and ROS formation. DNA fragmentation has also been reported in the sperm of convalescent COVID-19 patients (108–110).

Furthermore, maternal immune activation during pregnancy can have adverse effects on the cardiovascular system of the offspring and may potentiate the activation of inflammatory pathways in neonates and ROS formation (102). SARS-CoV-2 can influence mitochondrial damage in peripheral blood cells and cardiac cells, which can potentially affect oocyte mitochondria (59, 62, 63). However, the quality of oocytes in recovered patients has been reported as normal (111). Since maternal mitochondria are transferred to the zygote during fertilization and mitochondrial DNA mutations can predispose individuals to cardiovascular diseases, there may be risks associated with mitochondrial damage due to COVID-19 (112, 113).

## Conclusion

SARS-CoV-2's impact extends beyond the acute respiratory distress often spotlighted in medical narratives. Its ability to affect multiple organs and lead to serious cardiovascular complications provides a broader perspective on the virus's toll. As highlighted in our review, while the mechanisms underlying these complications are not fully defined, they likely involve hypoxia, endothelial dysfunction, and an exacerbated immune response. Notably, persistent cardiovascular ramifications have emerged not just in symptomatic COVID-19 survivors but also in those who remained asymptomatic.

With an astounding 756 million confirmed COVID-19 cases worldwide, the repercussions of long COVID, especially its cardiovascular sequelae, present both a challenge and a burden to global healthcare systems. We have reviewed several proposed pathophysiological mechanisms for these complications, including systemic biological aging, gut microbiome disruptions, mitochondrial and direct cardiac damage, sustained systemic inflammation, and epigenomic alterations.

Nevertheless, a significant gap remains in our comprehensive understanding of these mechanisms, with many still hypothesized and awaiting rigorous scientific validation. The urgency of the current situation underscores the need for continued research into the pathophysiology of long COVID. Only by elucidating these pathways can we hope to identify effective therapeutic targets and address the extensive cardiovascular consequences of this pandemic.
